# The (Lack of) DNA Double-Strand Break Repair Pathway Choice During V(D)J Recombination

**DOI:** 10.3389/fgene.2021.823943

**Published:** 2022-01-05

**Authors:** Alice Libri, Timea Marton, Ludovic Deriano

**Affiliations:** Genome Integrity, Immunity and Cancer Unit, Institut Pasteur, Université de Paris, INSERM U1223, Equipe Labellisée Ligue Contre Le Cancer, Paris, France

**Keywords:** DNA double-strand break, V(D)J recombination, non-homologous end-joining, homology-directed repair, DNA end resection, DNA double-strand break repair pathway choice

## Abstract

DNA double-strand breaks (DSBs) are highly toxic lesions that can be mended via several DNA repair pathways. Multiple factors can influence the choice and the restrictiveness of repair towards a given pathway in order to warrant the maintenance of genome integrity. During V(D)J recombination, RAG-induced DSBs are (almost) exclusively repaired by the non-homologous end-joining (NHEJ) pathway for the benefit of antigen receptor gene diversity. Here, we review the various parameters that constrain repair of RAG-generated DSBs to NHEJ, including the peculiarity of DNA DSB ends generated by the RAG nuclease, the establishment and maintenance of a post-cleavage synaptic complex, and the protection of DNA ends against resection and (micro)homology-directed repair. In this physiological context, we highlight that certain DSBs have limited DNA repair pathway choice options.

## Introductory Remarks

The integrity of a cell’s genome is continuously threatened by exogenous or endogenous factors generating DNA damage of various nature, which can impact a single nucleotide or result in lesions of the DNA backbone. Independently of the type or circumstances leading to the DNA damage, it must robustly be sensed, signaled, and repaired, ideally resulting in no or minimal alterations to the genetic code and recovery of an intact genome. Mammalian cells are equipped with several molecular tool kits warranting efficient repair of damaged DNA, where the nature of the DNA lesion largely dictates the selected repair apparatus. Nevertheless, multiple DNA repair pathways exist for a single type of damage such as the case for the mending of DNA double-strand breaks (DSBs). DNA DSBs are often considered as the most deleterious form of DNA damage for a cell, resulting in the physical separation of DNA molecules. Failure to accurately repair DSBs can lead to cell death or to DNA structural changes (*i.e.,* loss of genetic material, sequence alterations or joining of the wrong couple of DNA ends generating chromosomal translocations) potentially triggering carcinogenesis or onset of pathologies, including neurodegenerative diseases or immunodeficiencies (Mitelman et al., 2007; Jackson and Bartek, 2009; Ciccia and Elledge, 2010; Goldstein and Kastan, 2015). Maybe recklessly, chromosomal breakage has been co-opted by the immune system as an integral part of B- and T-cell development when V(D)J recombination–a programmed DNA rearrangement process–generates a vast array of antigen receptor molecules. V(D)J recombination is initiated when the lymphoid-restricted recombination-activating genes *RAG1* and *RAG2* are expressed and form a site-specific endonuclease (the RAG nuclease or RAG recombinase) that induces DSBs within T cell receptor (TCR, α/δ, β, γ) and Ig (h, κ, λ) gene loci. Despite the existence of multiple DSB repair pathways, including the canonical non-homologous end-joining (NHEJ) and homologous recombination (HR) pathways as well as additional (micro)homology-directed sub-pathways, RAG-initiated DSBs are “almost” exclusively repaired by NHEJ. In the following review, we address various parameters which restrict DNA DSB repair pathway choice in lymphocytes undergoing V(D)J recombination and discuss how NHEJ-mediated repair impacts on successful antigen receptor gene assembly or association to immunodeficiencies and lymphoid cancers.

## V(D)J Recombination and Double-Strand Break Repair

**FIGURE 1:**
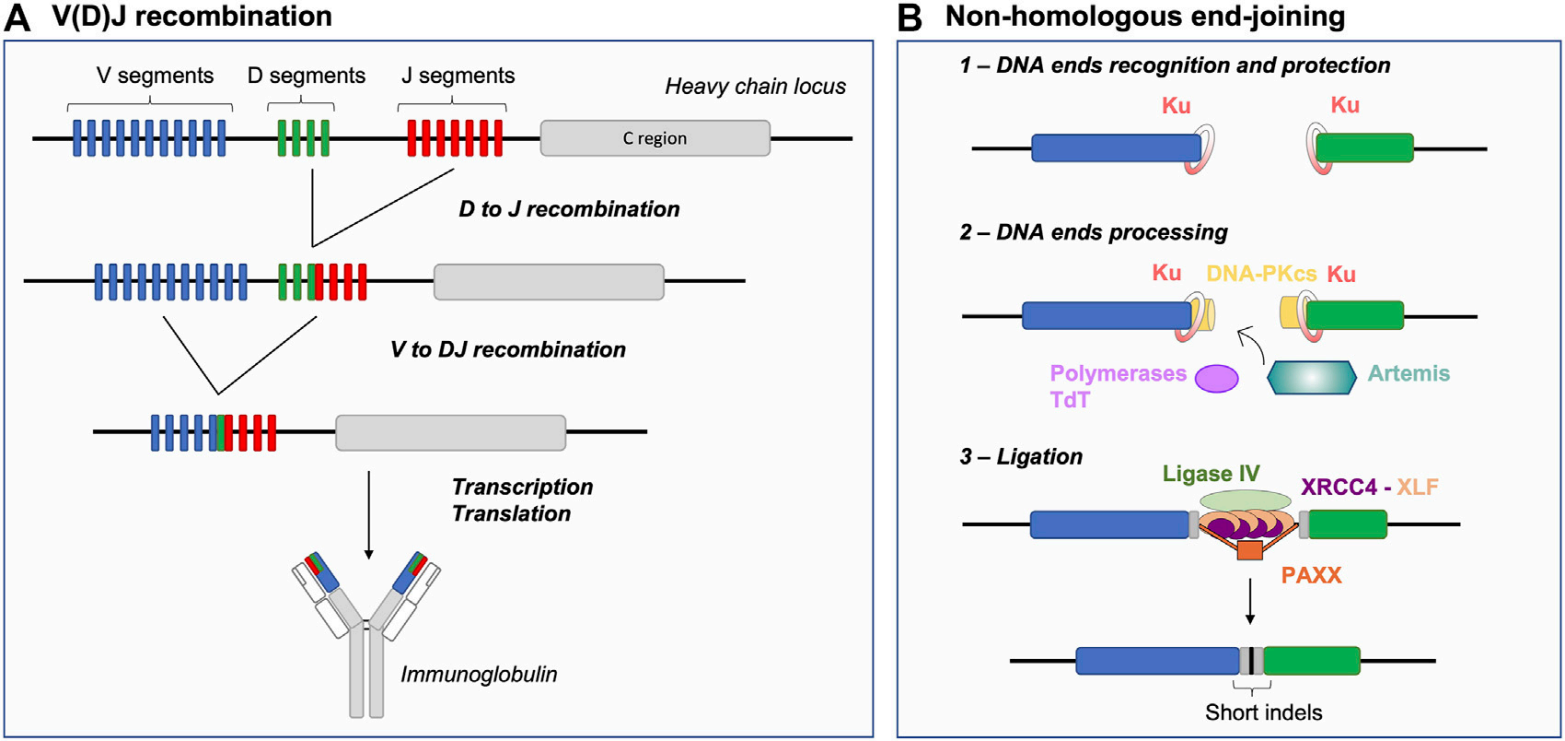
V(D)J recombination and NHEJ Basics: Generating antigen receptor diversity. **(A)** V(D)J recombination at the immunoglobulin heavy chain locus (depicted as an example) consists in a sequential 2-step rearrangement of V, D and J segments. This combinatorial process generates the diversity of antigen receptors. **(B)** After RAG cleavage, the NHEJ repair pathway is initiated by binding of the Ku70/80 heterodimer (Ku) to DNA ends. Ku together with DNA-PKcs form the DNA-PK holoenzyme. RAG DNA ends are then processed by the endonuclease Artemis and polymerases (*e.g.,* Pol µ), specifically the terminal deoxynucleotidyl transferase (TdT), resulting in increased junctional diversity (in gray). This additional diversity is generated, prior to joining, in two forms: 1) P- palindromic sequences, produced through the endonuclease action of Artemis at RAG-induced hairpin-sealed ends and 2) N-nucleotide sequences, the addition of non-templated nucleotides by TdT. Finally, the ligation complex composed of Ligase IV, XRCC4 and XLF joins the processed ends. Joining of DNA ends via NHEJ further participates to generating indels, moreover favoring junctional diversity. NHEJ: non-homologous end-joining, indels: insertions or deletions.

V(D)J recombination is a somatic antigen receptor gene rearrangement process occurring in developing B and T cells, involving rearrangement of V (variable), D (diversity) and J (joining) gene segments located within the Ig or TCR locus (Figure 1A) (reviewed in (Gellert, 2002; Litman et al., 2010; Schatz and Ji, 2011; Roth, 2014; Lescale and Deriano, 2016)). This locus-specific reaction is initiated by the RAG nuclease which introduces two DSBs at recombination signal sequences (RSSs) flanking selected V, D and J segments. RAG-DSBs pose a threat to overall genome stability and thus the activity of the RAG recombinase is tightly controlled (Roth, 2003; Roth, 2014; Lescale and Deriano, 2016).

Lymphocytes, as any other cell types, possess several DSB repair pathways including HR and NHEJ, which are considered the main DNA DSB repair pathways. HR is based on the capacity of the cellular machinery to find and access an intact template (sister chromatid or chromosome homolog) used to mediate error-free repair of the break. Initiation of HR involves the identification of broken DNA end(s), a 5′-3′ nucleolytic digestion process generating 3′ single-stranded DNA (ssDNA) (end resection) permitting homology search and DNA synthesis (Elbakry and Löbrich, 2021). NHEJ is thought to be a rapid and efficient way of repairing DSBs, as it involves the identification and (quasi) direct ligation of the two DNA ends without search for (extended) homology (Figure 1B) (reviewed in (Chang et al., 2017)). Briefly, upon detection of a DSB, the Ku70/80 heterodimer (Ku) is loaded onto DNA ends and acts as a scaffold for recruitment of additional NHEJ factors (Gottlieb and Jackson, 1993; Nick McElhinny et al., 2000; Walker et al., 2001; Fell and Schild-Poulter, 2015; Ochi et al., 2015). Ku recruits the DNA-dependent protein kinase catalytic subunit (DNA-PKcs) to form the DNA-PK holoenzyme that phosphorylates multiple substrates, promoting synapsis of DNA ends and facilitating the recruitment of end processing and ligation enzymes (Chen X. et al., 2021; Zha et al., 2021). The ligation complex, composed of Ligase IV-XRCC4-XLF, joins the ends together (Ahnesorg et al., 2006; Buck et al., 2006). PAXX, a paralog of XRCC4 and XLF, also contributes to end-joining during NHEJ (Ochi et al., 2015; Lescale et al., 2016b) notably by promoting accumulation of Ku at DSBs (Liu et al., 2017). This repair pathway, as opposed to HR, is sometimes defined as error-prone because it can generate small insertions and deletions (indels) (Figure 1B) (Stinson et al., 2020). DNA 5′ end resection is a major determining factor for the NHEJ to HR choice in cells, as mentioned above HR involves the formation of extensive 3′ ssDNA. The chromatin-bound protein 53BP1, together with downstream effectors, counteracts DNA end resection and thus act as a pro-NHEJ regulator upon DSB injury (Mirman and de Lange, 2020). Alternative end-joining (alt-EJ) and single-strand annealing (SSA) are yet other DSB repair pathways relying on intermediate length of DNA end resection and bias towards usage of (micro)homologies (Symington, 2016). These pathways are intrinsically unfaithful as they generate deletions between microhomology tracts and as alt-EJ is associated to genomic instability, notably chromosomal translocations (Corneo et al., 2007; Soulas-Sprauel et al., 2007; Yan et al., 2007). Alt-EJ is thought to be particularly active in cells deficient for HR or NHEJ (Corneo et al., 2007; Soulas-Sprauel et al., 2007; Yan et al., 2007; Wyatt et al., 2016), although some studies indicate that this pathway is also utilized in DNA repair proficient cells (Lee et al., 2004; Corneo et al., 2007; Coussens et al., 2013; Deriano and Roth, 2013).

During V(D)J recombination, antigen receptor gene diversity is achieved by 1) unique combinations of V, D and J coding segments, so called combinatorial diversity and 2) the imprecision of the DSB repair reaction at segment joints–driven by NHEJ and the action of the terminal deoxynucleotidyl transferase (TdT), termed junctional diversity (Figure 1B) (Gilfillan et al., 1993; Komori et al., 1993; Ramsden, 2011). NHEJ thus offers the ideal repair pathway to permit Ig and TCR gene diversification in early lymphocytes as opposed to resection- and homology-based repair pathways that would ultimately restore germline sequences or generate genetic instability in the cell. In the following sections, we address different parameters that limit DSB repair pathway choice to NHEJ during V(D)J recombination, including 1) the nature of DSB end structures, 2) the establishment of DSB end synapsis and 3) the impediment of DNA end resection and (micro)homology-driven repair.

## DNA End Structures–Meant to Be Repaired by Non-Homologous End-Joining

**FIGURE 2:**
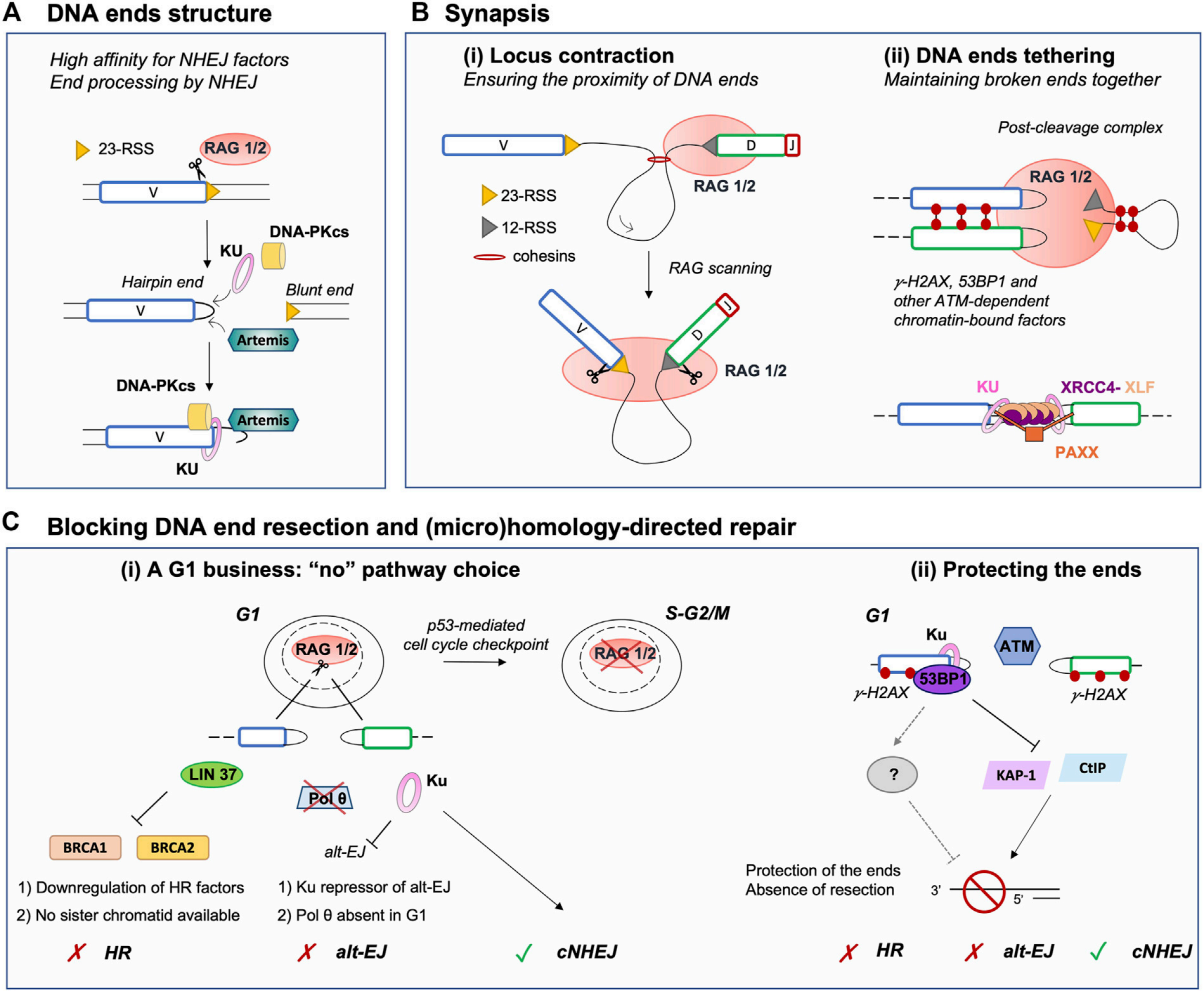
Major parameters restricting DNA repair pathway choice to NHEJ during V(D)J recombination. **(A)** RAG-induced breakage generates a covalently sealed hairpin end (coding end) and a blunt end (signal end). This facilitates the loading of Ku, which acts as a scaffold for other NHEJ factors, as it has a high affinity for blunt or hairpin sealed ends. In addition, hairpin sealed ends require to be opened by another NHEJ factor Artemis, which renders ends compatible for ligation. Thus, this DNA end topology contributes to the establishment of a NHEJ-prone environment. **(B) (i)** Upon binding an RSS, RAG scans the adjacent chromatin by a loop extrusion mechanism. Breakage is induced only upon reaching a compatible RSS, ensuring the induction of DSBs in close proximity despite the large size of the immunoglobulin locus. **(ii)** Following DSB induction, RAG remains bound to DNA ends in a post-cleavage complex (PCC). The PCC together with NHEJ and ATM-dependent chromatin-bound DNA factors (*e.g.,* phosphorylated H2AX and 53BP1) favor DNA ends tethering and stabilization. This likely prevents the search for distant partner DNA ends and channels broken DSB ends to NHEJ for safe repair. **(C) (i)** V(D)J recombination is a G1-restricted process, as RAG is degraded upon entry in the S phase. In G1, HR cannot operate as pre-replicative cells do not harbor a sister chromatid, used as a template for repair. In addition, several factors required for HR are transcriptionally repressed in G0/G1. Similarly, Pol θ, an important factor for alt-EJ, is poorly expressed in G1 consequently limiting the use of this repair pathway. Furthermore, alt-EJ is blocked by Ku upon binding DNA ends, yet again promoting processing and repair by NHEJ. **(ii)** Chromatin DSB-response factors γH2AX, 53BP1 and possibly additional downstream effectors contribute to the protection of RAG-DSB ends by blocking the activity of nucleases such as CtIP or acting via transcriptional repressors such as KAP-1. This protection prevents DNA end resection, an essential intermediate step for (micro)homology-directed repair (*e.g.,* alt-EJ, HR, etc.), hence promoting NHEJ. NHEJ: non-homologous end-joining, RSS: Recombination Signal Sequence, Alt-EJ: alternative end-joining, HR: homologous recombination.

Broken DNA ends often cannot be directly reattached and require processing prior to mending, thus the nature of the broken ends acts as an important factor influencing repair pathway choice (Chang et al., 2017). During V(D)J recombination, the RAG complex promotes the assembly of a pre-synaptic complex that includes a 12 and a 23 RSS prior to conducting its nuclease activity (Schatz and Ji, 2011). DNA cleavage occurs in two steps and relies on RAG1, RAG2, a divalent metal ion, and the ubiquitous bending factors HMGB1 or HMGB2. RAG introduces a nick between each RSS and its flanking coding sequence, generating a free 3′-OH group which then attacks the opposite strand by transesterification. This cleavage reaction results in four broken DNA ends with specific structures: two hairpin-sealed coding ends (CE) at gene segments and two blunt signal ends (SE) at RSSs (Figure 2A). Upon cleavage, RAG-induced DNA breaks activate Ataxia telangiectasia mutated (ATM), an important mediator of the DNA DSB response (Helmink and Sleckman, 2012). Activated ATM phosphorylates numerous proteins that promote the G1/S checkpoint and participate in DNA end protection (see below), favoring NHEJ. Ku has a strong affinity for hairpin sealed, blunt or short overhang DNA ends (Falzon et al., 1993; Downs and Jackson, 2004), directing RAG-DSBs towards NHEJ repair. Ku not only binds avidly broken ends but also serves as a scaffold for the recruitment of DNA-PKcs, forming the DNA-PK holoenzyme, and downstream NHEJ factors that permit the processing and ligation of RAG-induced DSB ends. This second attribute of Ku is particularly important for CEs as blunt SEs can be directly ligated by XRCC4-Ligase IV to form DNA circles (*i.e.,* in the case of deletional recombination). Indeed, CEs necessitate the action of the endonuclease Artemis to open the hairpin structure (Figure 2A). Proper Artemis endonuclease activity requires DNA-PK and leads to the formation of protruding 3′ ends with an -OH group (Ma et al., 2002). This latter DNA end topology favors repair by NHEJ, as XRCC4-Ligase IV necessitate a -OH at both DNA ends for ligation. Additionally, the XRCC4-Ligase IV complex can stimulate the removal of few nucleotides-long overhangs–generated by Artemis–prior to ligation (Gerodimos et al., 2017). Notably, Ku also promotes the recruitment of TdT–the third lymphoid-specific protein in addition to RAG1 and RAG2 – that adds nucleotides at Artemis-opened CEs preceding the ligation by XRCC4-Ligase IV. This links NHEJ to the generation of junctional diversity at coding joins (Gilfillan et al., 1993; Komori et al., 1993; Purugganan et al., 2001; Ramsden, 2011), increasing the genetic diversity of V(D)J rearrangement outcomes. Therefore, the topology of RAG-induced DSB ends significantly biases repair towards NHEJ, by generating an NHEJ prone environment.

## Synapsis - Keeping DNA Ends Together for Safe Repair by Non-Homologous End-Joining

Maintaining broken DNA ends in close proximity is a major parameter that influences pathway choice, notably because NHEJ requires the physical proximity of both DNA ends, while it is dispensable for certain HR reactions such as break-induced replication (Pham et al., 2021). Synapsis of DSB ends during V(D)J recombination is quite challenging as it involves the sequestration of four DSB ends (*i.e.,* two CEs and two SEs). Additionally, V(D)J recombination implicates gene segments that can be situated at considerable distances from one another; for instance the murine germline immunoglobulin heavy chain (IgH) locus spans approximately 2.75 Mbp of chromosome 12 (Lucas et al., 2015). For efficient V(D)J recombination, 1) V, D and J gene segments must be brought in vicinity of each other prior to cleavage and 2) DNA ends, specifically CEs which require processing, must be kept together for ligation.

Contraction and spatial reorganization of antigen receptor loci during V(D)J recombination rely largely on the formation of chromatin loops through a cohesin-dependent extrusion process (Bossen et al., 2012; Ebert et al., 2015; Ba et al., 2020; Hill et al., 2020; Dai et al., 2021; Davidson and Peters, 2021), as well as transcription and subnuclear relocation (Rogers et al., 2021). Remarkably, this mechanism poises the loci for recombination independently of RAG, but also endows RSS-bound RAG with the ability to scan chromatin for a partner RSS, providing directionality and spatial restriction to RAG activity within the chromatin loop domain (Figure 2Bi) (Lin et al., 2018; Zhang et al., 2019; Ba et al., 2020; Hill et al., 2020; Dai et al., 2021). Additional chromatin-bound factors such as 53BP1 contribute to bringing V(D)J segments close-by, as depletion of this latter factor results in a reduction of distal V to DJ segments joins (Difilippantonio et al., 2008). Induction of two DSBs in the vicinity of one another likely contributes to favoring rapid repair of RAG-induced DNA breaks by NHEJ, without the need to search for partner DNA ends.

After cleavage, the RAG proteins stay associated with the DNA ends in a post-cleavage complex (PCC) (Figure 2Bii). Mutations resulting in RAG-PCC destabilization were shown to increase repair of RAG-mediated DSBs via HR and alt-EJ (Lee et al., 2004; Corneo et al., 2007; Coussens et al., 2013; Deriano and Roth, 2013), pathways considered as unconventional for V(D)J recombination. These observations suggested that the RAG-PCC might contribute to shepherding DNA ends to the NHEJ machinery for repair, thus protecting them from error prone end-joining pathways and aberrant recombination events (Roth, 2003; Lee et al., 2004; Deriano and Roth, 2013). Indeed, a RAG2 mutant - possessing deletion of C-terminal residues 352–527 (core RAG2) - destabilizes the RAG-PCC and is associated with an increased rate of aberrant recombination outcomes *in vitro* and to inter-chromosomal translocations involving the V(D)J loci *in vivo* (Sekiguchi et al., 2001; Talukder et al., 2004; Corneo et al., 2007; Curry and Schlissel, 2008; Deriano et al., 2011; Coussens et al., 2013). Additionally, core RAG2/p53-deficient mice present increased genomic instability and accelerated lymphomagenesis via alt-EJ, generating tumors bearing a complex landscape of chromosomal rearrangements (Deriano et al., 2011; Mijušković et al., 2012; Mijušković et al., 2015). Strikingly, the lymphomas and translocations observed in the latter animals resemble those of ATM-deficient mice, suggesting that a similar DNA end destabilization mechanism might underlie genomic instability and lymphomagenesis in both mouse models (Deriano et al., 2011). Consistent with this, ATM - beyond its role in activating checkpoints - is important for the stability of RAG-PCCs *in vivo* (Bredemeyer et al., 2006). Upon DSB damage, ATM phosphorylates chromatin- and DNA-associated proteins, including the histone variant H2AX (forming γH2AX), 53BP1, MDC1 and factors of the MRN complex (MRE11, RAD50, and NBS1) that assemble on both sides of DNA breaks forming so-called nuclear DNA repair foci. The stabilization function of ATM depends on its kinase activity. Thus, formation of ATM-dependent DNA repair foci has been proposed to tether DNA ends for proper joining via NHEJ (Figure 2Bii). In ATM-deficient cells undergoing V(D)J recombination, the fraction of CEs which evade the PCC are occasionally joined aberrantly, forming chromosomal deletions, inversions, and translocations (Bredemeyer et al., 2006; Helmink and Sleckman, 2012). Altogether, these results indicate that RAG2 (by extension the RAG-PCC) and ATM share mechanistic properties during V(D)J recombination, via the stabilization of broken DNA-ends consequently avert the use of alternative repair pathways.

Additional insights into the mechanisms responsible for the stabilization of RAG-cleaved DNA ends come from the analysis of animal models double-deficient for XLF and ATM or core RAG2. XLF and XRCC4 are two distantly related members of the same protein family and share structural similarity (Callebaut et al., 2006; Andres et al., 2007; Li Y. et al., 2008). Together, they form long filaments, thought to help DNA end tethering and synapsis during repair (Figure 2Bii) (Tsai et al., 2007; Riballo et al., 2009; Hammel et al., 2011; Ropars et al., 2011; Reid et al., 2015; Chen S. et al., 2021). In contrast to other NHEJ-deficient mice, XLF-deficient mice are not markedly immune-deficient and early lymphoid cells from these animals perform nearly normal V(D)J recombination. These observations suggest that other factors or pathways compensate for XLF function during V(D)J recombination (Li G. et al., 2008). In fact, cells deficient for both XLF and ATM-dependent DSB response (*e.g.,* XLF and ATM, 53BP1, or H2AX double mutants) display severe block in lymphocyte development and a significant defect in the repair of RAG-mediated DSBs. This reveals functional redundancy between XLF and ATM-DSB response factors during V(D)J recombination (Li G. et al., 2008; Zha et al., 2011; Liu et al., 2012; Oksenych et al., 2012; Oksenych et al., 2013; Vera et al., 2013; Kumar et al., 2014). Similarly, core RAG2/XLF double deficiency leads to a profound lymphopenia associated with a severe defect in joining of RAG-cleaved DNA ends (Lescale et al., 2016a). These findings are consistent with a two-tier model in which the RAG proteins, together with the ATM chromatin DSB-response, collaborate with NHEJ factors to promote functional V(D)J recombination and emphasize the importance of DNA end tethering for proper repair.

## Blocking DNA End Resection and (micro)Homology-Driven Double-Strand Break Repair

### A G1-Phase Business

RAG-induced V(D)J recombination is limited to the G1 phase of the cell cycle, which offers an additional level of restriction to NHEJ-driven repair. This is due to the specific destruction of RAG2 during the G1-to-S transition that is triggered by phosphorylation of the T490 residue (Li et al., 1996). Additionally, RAG-induced DSBs trigger an ATM/p53-dependent DSB response that promotes G1/S cell cycle arrest and eventually cell death (Figure 2Ci). Finally, RAG-DSBs activate a specific checkpoint that opposes the pre-B cell receptor proliferative signals and prevent cells from entering into S phase before resolving the damage (Bredemeyer et al., 2008; Bednarski and Sleckman, 2012; Bednarski et al., 2016).

As the ideal template for HR is the sister chromatid, HR is restricted to the S and G2 phases of the cell cycle and cannot fully operate in G1-phase cells. Alt-EJ (and SSA) do not possess this constraint, thus could potentially serve as alternatives to NHEJ for repair of DSBs in non-dividing cells. Using high-throughput sequencing techniques, it was recently shown that end joining of RAG-induced DSBs is virtually null in G0/G1-arrested progenitor (pro-) B cells deficient for XRCC4 (Yu et al., 2020). Within the same setting, Cas9-induced DSBs are also poorly repaired, suggesting that additional factors, other than RAG, limit the access of broken DNA ends to alt-EJ pathways in G0/G1-phase cells. Similarly, DSBs generated by RAG, Cas9 or zinc finger endonucleases in G0/G1-arrested pro-B cells remain unjoined in the absence of Ligase IV (Liang et al., 2021). However, Ku70-deficient or Ku70/Ligase IV-deficient G0/G1-arrested pro-B cells perform quite robust end-joining, albeit at lower levels than wild type cells, indicating that Ku acts as a strong repressor of alt-EJ in G0/G1-phase cells (Figure 2Ci) (Frock et al., 2021; Liang et al., 2021). Cells might also not be fully equipped to perform resection- and homology-dependent repair in G0/G1. For instance, LIN37, a component of the DREAM transcriptional repressor, inhibits resection and HR in G0/G1-blocked pro-B cells by repressing the expression of HR proteins such as BRCA1, BRCA2, PALB2 and RAD51 (Chen B. R. et al., 2021). Similarly, DNA polymerase theta (Pol θ, encoded by *Polq* in mice), implicated in alt-EJ (Ramsden et al., 2021), is not expressed in G0/G1-arrested pro-B cells (Figure 2Ci) (Yu et al., 2020).

Nevertheless, analysis of mice harboring combined deficiency in p53 and in NHEJ (*i.e.,* Ku, XRCC4 or Ligase IV) irremediably develop aggressive pro-B lymphomas displaying RAG-dependent translocations and amplifications between *Igh* and *c-Myc* by alt-EJ (Nussenzweig and Nussenzweig, 2010; Gostissa et al., 2011; Ramsden and Nussenzweig, 2021). It was suggested that p53 deficiency enables cells to move inappropriately into S phase and acquire DSBs that initiate chromosomal translocations and amplifications (Paulson et al., 1998; Zhu et al., 2002). In fact, an *in vitro* study using XRCC4/p53-deficient pro-B cell lines shows that the transition from G0/G1-phase to S-G2/M-phases of the cell cycle enables alt-EJ repair, promoting massive genetic instability in the form of chromosomal deletions and translocations (Yu et al., 2020). It is tempting to speculate that unrepaired G1-DNA breaks progressing to S-G2/M get lost in the cellular space with unprotected DNA ends being subjected to repetitive nuclease attacks until (micro)homology-driven alt-EJ stabilizes them in *cis* or *trans*. In that regard, although multiple homology-directed sub-pathways would theoretically be able to process these lost DNA ends (Elbakry and Löbrich, 2021), the repair of G0/G1-DSBs in S-G2/M would strictly depend on Pol θ. Indeed, in XRCC4/Pol θ/p53-triple deficient pro-B cells, DSBs induced in G1 accumulate in the form of chromosomal breaks resulting in lethality at the next mitosis (Yu et al., 2020). Whether Pol θ contributes to the development of pro-B cell lymphomas, carrying *Igh/c-Myc* translocations, in NHEJ/p53-deficient animals remains to be addressed.

### DNA End Protection

The extent of resection is actively regulated by the protection of DNA ends, which limits the access of nucleases to the break sites. In addition to the above-mentioned parameters (*i.e.,* DNA end structures and synapsis), several DSB response chromatin-bound factors localize at RAG-DSBs and are thought to protect DNA ends against resection, including γH2AX and 53BP1 (Difilippantonio et al., 2008; Helmink et al., 2011; Zha et al., 2011; Dorsett et al., 2014; Lescale et al., 2016a; Chen B. R. et al., 2021). In G1, yH2AX prevents CtIP-mediated nucleolytic resection (Helmink et al., 2011). Similarly, KAP-1, a transcriptional repressor modulating chromatin structure, was shown to promote resection in G1 lymphocytes in the absence of yH2AX and 53BP1 (Tubbs et al., 2014). Moreover, depletion of 53BP1 in Ligase IV-deficient G0/G1-blocked pro-B cells results in increased levels of resection at irradiation-induced DSB ends, demonstrating that 53BP1 is crucial for DNA end protection in this cell-cycle phase (Figure 2Cii) (Chen B. R. et al., 2021). The Shieldin complex, composed of SHLD1, SHLD2, SHLD3 and MAD2L2/REV7, acts downstream of 53BP1-RIF1 to antagonize DNA end resection and favor NHEJ over HR (Greenberg, 2018; Setiaputra and Durocher, 2019; Mirman and de Lange, 2020; de Krijger et al., 2021). It acts in a paradoxical manner as it requires to bind >50 nt-long ssDNA ends in order to hinder DNA end resection (Dev et al., 2018; Findlay et al., 2018; Gao et al., 2018; Noordermeer et al., 2018). Mechanistically, it is thought to directly inhibit resection by physically blocking access of nucleases to the free ssDNA-dsDNA ends. Additionally, this complex promotes the recruitment and coordination of additional factors leading to the processing of ssDNA-dsDNA intermediates prior to NHEJ repair such as ASTE1, which cleaves the protruding ssDNA, and the CST (CTC1–STN1–TEN1)–DNA polymerase-α–primase complex, to fill in the residual ssDNA (Mirman et al., 2018; Zhao et al., 2021). Although the Shieldin complex counteracts HR in BRCA1-deficient cells and is important for NHEJ-driven repair during class switch recombination or in the fusion of unprotected telomeres, it seems dispensable for V(D)J recombination (Dev et al., 2018; Ghezraoui et al., 2018; Mirman et al., 2018; Ling et al., 2020). Indeed, SHLD2 or REV7 deficiencies in mice do not significantly alter lymphocyte development and V(D)J recombination (Ghezraoui et al., 2018; Ling et al., 2020). It is to note that in wild-type cells, the processing of RAG-induced DNA ends does not generate >50 nt-long ssDNA intermediates, thus potentially explaining why Shieldin-mediated protection prior to joining seems dispensable during V(D)J recombination. However, whether the Shieldin complex plays a role in protecting RAG-generated DNA ends against resection in the context of crippled NHEJ remains to be investigated. In XLF-deficient mice (impaired NHEJ), 53BP1 plays an essential role in counteracting resection at RAG-DSB ends, promoting V(D)J recombination and lymphocyte differentiation (Liu et al., 2012; Oksenych et al., 2012). Nonetheless, it is unclear if this DNA end protection is mediated through 53BP1 downstream effectors (*e.g*., Shieldin complex) or via intrinsic properties of 53BP1 (Figure 2Cii). Notably, Ku also antagonizes DNA end resection through at least two distinct mechanisms 1) by blocking the access of nucleases to DSB ends (Zahid et al., 2021) and 2) by recruiting TdT which promotes template-independent and -dependent synthesis prior to ligation (Loc’h and Delarue, 2018). Taken together, in the context of V(D)J recombination the downregulation of the DSB end resection machinery and the protection of DNA ends by chromatin-bound factors and Ku seem to act as forefront anti-resection barriers, promoting repair via NHEJ but not HR or alt-EJ (Figure 2C).

## Conclusion and Perspectives

In this review, we present V(D)J recombination as a relevant biological setting to investigate factors influencing DSB repair pathway choice, specifically those constraining the repair of DSBs to NHEJ. This repair pathway is essential for V(D)J combinatorial rearrangement as well as for the generation of diversity at V(D)J junctions, two pre-requisites for antigen receptor gene diversification and the establishment of a primary immune repertoire. During V(D)J recombination, several factors prime for repair via NHEJ, including the spatial organization of the genomic loci subjected to these rearrangement events. Additionally, the RAG nuclease, the type of generated DSBs, the G1 phase-specific environment and the dedicated DSB response predispose (arguably dictate) repair through NHEJ. Albeit numerous the studies which shed light onto the mechanisms through which DSBs generated during V(D)J recombination are biased towards repair by NHEJ, several questions remain unanswered. For instance, while the RAG-PCC plays a role in favoring repair via NHEJ, possibly by stabilizing DNA ends, it remains unclear if the RAG proteins directly interact with certain NHEJ factors and whether such interaction(s) would contribute to NHEJ pathway choice. We also discussed the importance of blocking DNA end resection during V(D)J recombination through the action of specific chromatin DSB response factors, most notably 53BP1. Whether this end protection only relies on the capacity of the chromatin-bound factors to maintain a stable PCC or whether it also requires specific downstream effectors to act at the DSB ends is unclear. In that regard, it is interesting to note that the mode of action of the Shieldin complex on DSB ends generated by AID during IgH class switch recombination is somewhat reminiscent to that of Ku during V(D)J recombination. Both Ku and the Shieldin complex have the capacity to physically obstruct resection at DSB ends and to actively recruit factors implicated in DNA end modifications (*i.e.,* action of Ku and TdT/Artemis versus Shieldin-complex and ASTE1/CST-DNA polymerase α). In Ku-deficient G0/G1-arrested pro-B cells (and to a much lesser extent in XRCC4- or Ligase IV-deficient cells), V(D)J joints harbor rather short resection tracks (typically less than 100 nucleotides) (Yu et al., 2020; Liang et al., 2021). Could RAG-DSB ends benefit from Shieldin complex protection against resection in such circumstances? Additionally, the nature of alt-EJ and the factors implicated in alt-EJ in G0/G1-phase cells as opposed to Pol θ-mediated alt-EJ in S-G2/M remain to be established. The role of these sub-pathways in the onset of pro-B cell lymphomas in NHEJ/p53-deficient animals also remains to be investigated. Finally, antigen receptor loci relocate from the nuclear periphery to permissive euchromatin in the nuclear interior before V(D)J recombination (Rogers et al., 2021). This subnuclear relocation likely provides specific local chromatin environments that might influence downstream DSB repair events (Mitrentsi et al., 2020). Recent studies have also highlighted the importance of 3D genome (re)organization and dynamics in DSB repair, for instance through the establishment of γH2AX/53BP1 DSB response foci (Arnould et al., 2021) or the restriction of homology search during HR (Piazza et al., 2021), two crucial chromatin events influencing DSB repair outcome and pathway choice. How such chromosome dynamics contributes to (lack of) DNA DSB pathway choice and overall genome integrity maintenance during V(D)J recombination remains a question for future studies.
